# Imaging modalities in synchronous oligometastatic prostate cancer

**DOI:** 10.1007/s00345-018-2416-2

**Published:** 2018-08-01

**Authors:** Jurgen J. Futterer, Cristian Surcel, Roderick van den Bergh, Hendrik Borgmann, Alberto Briganti, Giorgio Gandaglia, Alexander Kretschmer, Piet Ost, Prasanna Sooriakumaran, Derya Tilki, Massimo Valerio, Guillaume Ploussard, Pieter J. L. De Visschere, Igor Tsaur, the EAU-YAU Prostate Cancer Working Party

**Affiliations:** 1 Department of Radiology and Nuclear Medicine, Radboudumc, P.O. Box 9101, internal postal code 766, 6500 HB Nijmegen, The Netherlands; 2 Department of Urology, Fundeni Clinical Institute, Carol Davila University of Medicine, Bucharest, Romania; 3 Department of Urology, University of Utrecht, Utrecht, The Netherlands; 4 Department of Urology and Pediatric Urology, University Medicine Mainz, Mainz, Germany; 5 Department of Urology, Urological Research Institute, Vita-Salute University and San Raffaele Hospital, Milan, Italy; 6 Department of Urology, Ludwig-Maximilians-University of Munich, Munich, Germany; 7 Department of Radiation Oncology and Experimental Cancer Research, Ghent University Hospital, Ghent, Belgium; 8 Department of Uro-oncology, University College London Hospital, London, UK; 9 Martini-Klinik Prostate Cancer Center, University Hospital Hamburg-Eppendorf, Hamburg, Germany; 10 Department of Urology, Centre Hospitalier Universitaire Vaudois, Lausanne, Switzerland; 11 Department of Urology, Saint Jean Languedoc Hospital, Toulouse, France; 12 Department of Radiology and Nuclear Medicine, Ghent University Hospital, Ghent, Belgium; 13 Department of Urology, University Hospital Hamburg-Eppendorf, Hamburg, Germany

**Keywords:** Prostate cancer, Oligometastatic, Imaging, PET-CT, PSMA, MRI

## Abstract

**Purpose:**

Along with a number of other malignancies, the term “oligometastatic” prostate cancer has recently emerged. It represents an attempt to define a subtype of cancer with a limited metastatic load that might perform more favorably than a distinctly disseminated disease, or even one that may be managed in a potentially curative way. Since there is currently a knowledge gap of what imaging modalities should be utilized to classify patients as having this type of tumor, we aimed to shed light on the role of conventional and marker-based imaging in the setting of synchronous oligometastatic prostate cancer as well as summarize the available evidence for its clinical application.

**Methods:**

A literature search on December 15th 2017 was conducted using the Pubmed database.

**Results:**

Functional imaging techniques like ^68^Ga PSMA. ^68^Ga PSMA PET-CT has currently been shown the best detection rates for the assessment of nodal, bone and visceral metastases, especially for smaller lesions at low PSA levels.

**Conclusions:**

Functional imaging helps detect low-burden disease metastatic patients. However, these imaging modalities are not available in every center and thus clinicians may be prone to prescribe systemic treatment rather than referring patients for cytoreductive treatments. We hope that the ongoing prospective trials will help guide clinicians in making a more personalized management of synchronous metastatic patients.

## Introduction

A total of 416,700 new cases of prostate cancer (PCa) were estimated to occur in Europe in 2012, while 92,200 males would succumb, making PCa besides non-melanoma skin cancers the most frequently diagnosed malignancy and at the same time the third leading cause of cancer death in men [1]. In non-metastatic disease, curative risk adapted approaches incorporating life expectancy, comorbidities and treatment preference, and at the same time accounting for the presumed clinical stage of the tumor, are offered for patients to choose between active surveillance, focal therapy, radical prostatectomy (RP) or radiation therapy (RT) accompanied by excellent survival probabilities [2].

Fortunately, only less than 1 out of 20 males with PCa is nowadays diagnosed with a de novo systemically disseminated cancer due to a wide adoption of PSA screening [3]. Once metastasized, primarily or progressing after local treatment, disease is, however, believed incurable and only manageable with palliative intent. Since Huggins and Hodges determined the hormonal dependence of PCa in the 1940s [4], androgen deprivation therapy (ADT) has been the frontline treatment for metastatic disease. However, disease progression to castration-resistant PCa (CRPCa) inevitably occurs after 2–3 years of ADT [5].

In general, disease classification as “metastatic” as opposed to “clinically localized” relies on the presence of metastatic lesions on conventional imaging, i.e., lymphatic or visceral metastases on computed tomography (CT) or magnetic resonance imaging (MRI) as well as osseous metastases on bone scan in accordance with recommendations of the European Association of Urology on imaging for disease staging [6]. In the past, men with metastatic disease were usually deprived of a local treatment (and subjected to ADT) under the assumption that this therapy may not exert any positive influence on the following course of the disease and may expose patients to unnecessary treatment-related morbidity. Consequently, it was carried out solely in highly selected cases experiencing local complications. Common practice was, e.g., the abandonment of RP if pelvic lymph node dissection demonstrated lymphatic metastasis on frozen section analysis. However, evidence emerged later that patients with positive nodes and complete RP have a survival advantage over their counterparts with abandoned RP [7, 8]. Thus, a number of patients with limited extent of disseminated disease might still benefit from local treatment (RP or RT), not only in terms of its alleviating local symptoms as a palliative care but also by experiencing a survival advantage (if not a cure) via a multimodal approach [9–11].

Ongoing challenge for a treating physician hence consists of identifying patients whose disease has progressed systemically, but in whom a local therapy combined with metastasis-directed and/or adjuvant systemic therapy might be possible. In this context, the term “oligometastatic disease” represents an attempt to define such a population of PCa patients. In view of a virtual revolution achieved in the last years in the area of molecular imaging modalities in PCa allowing for an improved sensitivity and specificity of metastasis detection in small-volume disease, it is apparent that the numbers of patients detected with a low-burden disseminated cancer as compared to localized disease will rise in the future, since occult lesions missed with conventional imaging may be better visualized with these novel methods.

Since there is neither unanimous consent on how to exactly classify this disease nor what imaging should be utilized for its definition, we strived to review the available evidence on conventional and molecular imaging modalities and their current and possible future clinical implication in the setting of the synchronous oligometastatic PCa (OPCa).

## Oligometastatic disease

Oligometastasis was first described by Hellmann and Weichselbaum in 1995, as they postulated that tumor progression occurs in a multistep fashion during its clinical evolution and the number and sites of metastatic lesions may reflect the state of tumor development [12]. According to this theory, there are intermediate cancer stages between purely localized and widely metastatic. Tumors in their early state of progression possess only a low dissemination capacity and hence lead to a few lesions limited to organs with the highest host receptivity offering optimal conditions for growth, proliferation and angiogenesis and where these cells are with a fragile invasive behavior may survive. Following this, malignant seeding capacity intensifies and promotes a multifocal dissemination. The authors also proposed that some patients bearing oligometastatic disease might be treated with curative intent.

In 2004, Singh et al. [13] were the first to use the term “oligometastatic disease” in the setting of PCa. They reported that PCa patients, who were initially treated with RT for localized/locally advanced disease and later developed OPCa (defined as ≤ 5 lesions; metachronous oligometastasis), had a superior survival over their counterparts with a more extended dissemination. They proposed to treat patients with OPCa aggressively to counteract further systemic seeding from metastatic lesions and improve long-term survival.

Traditionally, OPCa is referred to as a low-volume tumor burden up to 3–5 metastases in bone and/or lymph nodes on conventional imaging [3, 14]. Of note, novel imaging modalities such as ^11^C-Choline, ^11^C-Acetate, ^18^F-FDG or ^18^F-Choline PET-CT have also been used to classify patients as having OPCa [15–18]. Importantly, it is still unclear whether OPCa is simply a transitory phenomenon from clinically localized to polymetastatic cancer and shares similar molecular features with a high-burden disease [14]. Alternatively, the underlying biology at the molecular level in OPCa cells might be different to polymetastatic disease. Preclinical data suggest that oligometastatic progression of tumors is substantially driven by epigenetic alterations and, in particular, miRNAs, whereas polymetastatic dissemination is characterized by overexpression of genes associated with cell division and cell cycle progression [19–21]. This scenario might also hold true for PCa. Further comprehensive research in PCa is urgently needed to elucidate if there are really different molecular mechanisms between the true oligometastatic and pseudo-oligometastatic disease, which has already disseminated to a considerable extent with occult lesions, to facilitate selection of patients for local and/or metastases-targeted and/or adjuvant systemic therapy vs. palliative systemic treatment [14]. In addition, molecular classifiers are welcomed to pigeonhole OPCa patients with a predominantly androgen receptor-driven disease and for whom ADT or abiraterone would be an effective treatment as an adjuvant approach as opposed to those who might progress rapidly to CRPC. In particular, there might be some histological subtypes of PCa, such as ductal or neuroendocrine/small cell variants, which might benefit most from chemotherapy even in the oligometastatic state [22].

## CT and MRI

Both CT and MRI are the mainstay of whole-body morphological imaging; functional techniques in MRI provide additive data [23]. The imaging depicted metastatic state is key to patient management for biomarker development and for therapeutic clinical trials [24]. The literature shows a broad range in the diagnostic performance of both unenhanced CT and MRI in the detection of lymph node metastases. CT and MRI demonstrate an equally poor performance in the detection of lymph node metastases from PCa. Reliance on either CT or MRI will misrepresent the patient’s true status regarding nodal metastases, and thus misdirect the therapeutic strategies offered to the patient [25].

**Fig. 1:**
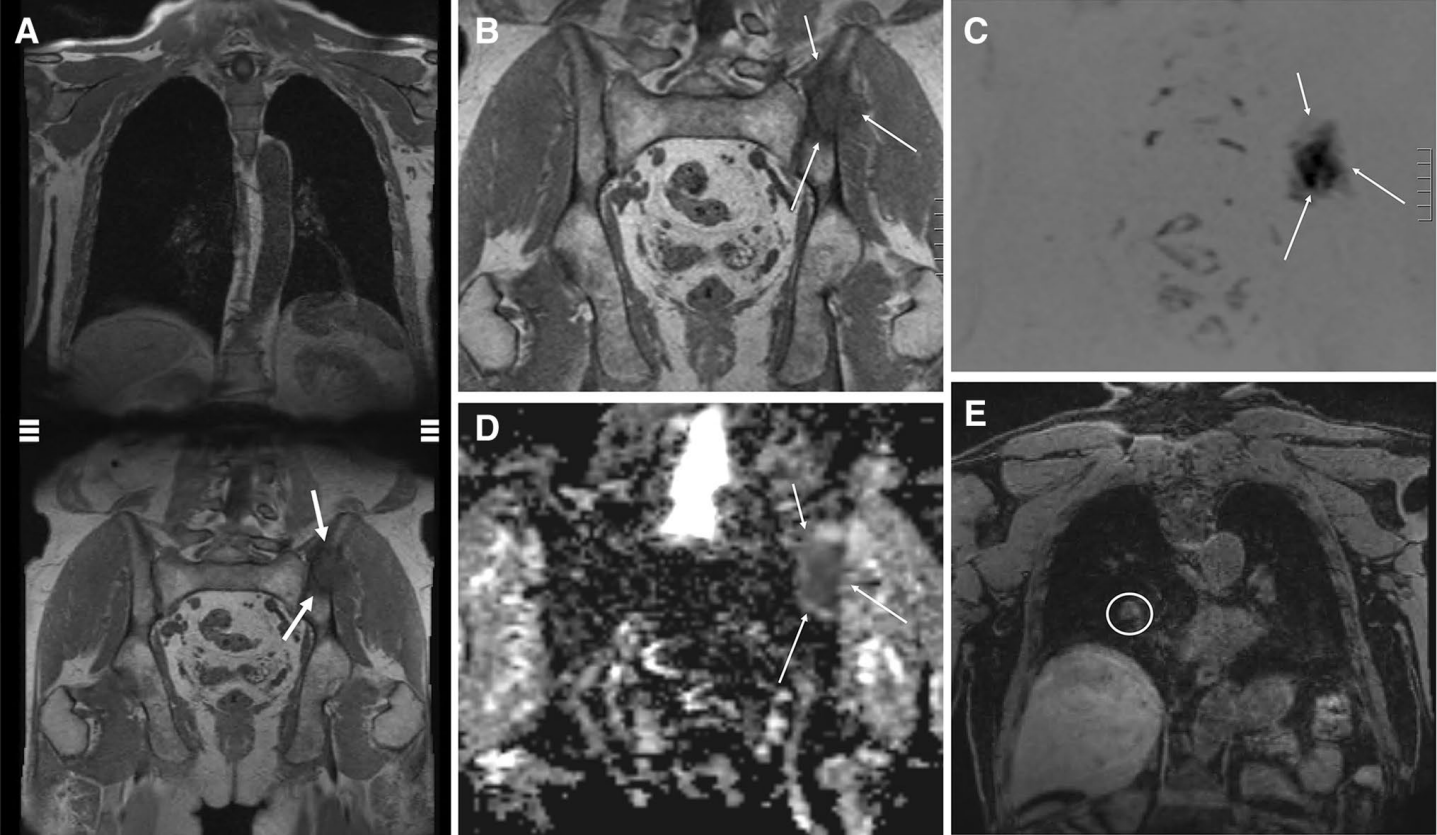
73-year-old man with a Gleason 4 + 3 PCa pT3 with PSA of 18.2 ng/ml. A whole-body MR examination was performed. Coronal whole body T1-weighted image (**a**) shows a left iliac bone metastasis (**b**; magnification; arrows). The bone metastasis (arrows) are evident on whole-body DWI images (**c**, **d**). **e** In the right lung, para-hilar, a 15 mm metastasis was observed (circle)

Whole-body MRI (WB-MRI) with conventional T1, T2 and inversion recovery sequences provides high tissue contrast for bone metastasis detection [26, 27]. The addition of diffusion-weighted imaging (DWI) to WB-MRI enables the study of extra-skeletal involvement, including lymph nodes and other soft-tissue metastases, without requiring intravenous contrast agents [28, 29]. The reported sensitivity and specificity for WB-MRI ranges from 98 to 100% and 98 to 100%, respectively. Inter-observer agreement for reading of WB-MRI images that include DW is very good (*K* = 0.87 [0.66; 1.00]) [27]. Furthermore, DWI in WB-MRI will allow quantification of tumor load [30]. The METastasis Reporting and Data System for Prostate Cancer (MET-RADS-P) imaging recommendations are designed to promote standardization and diminish variations in the acquisition, interpretation, and reporting of WB-MRI in advanced prostate cancer. The MET-RADS-P system provides comprehensive characterization of advanced PCa state, not only at the start of treatments but also over time as the disease evolves [30] (Fig. 1).

## Bone scan

For the staging of bone metastases, ^99m^Technetium-methylene diphosphonate bone scintigraphy (‘bone scan’) is the traditional established imaging modality. In patients with primary PCa, the current guidelines recommend a bone scan if there are symptoms suggestive of bone metastasis, or in asymptomatic patients when the PSA level is > 10 ng/ml, Gleason score ≥ 8 or clinical stage ≥ T3 (i.e., intermediate- and high-risk tumors) [6]. Bone scan has the disadvantage that it only assesses the skeleton and that it is hampered by a large number of inconclusive results warranting additional imaging with radiography, CT or MRI in about 16% of the cases [31, 32]. A bone scan can readily detect polymetastatic disease in symptomatic patients or in patients with PSA levels of ≥ 50 ng/ml with reported sensitivities of 78–95%, specificities of 72–100%, positive predictive value of 96% and negative predictive value of 60% [32, 33]. In asymptomatic patients the diagnostic yield is highly dependent on the PSA level, with positivity rates of only 2.3% for PSA levels of 0–9.9 ng/ml, 5.3% for PSA levels of 10–19.9 ng/ml and 16.2% for PSA levels of 20–49.9 ng/ml [34]. In recent years, with the advent of whole-body MRI and PET-CT as new comparators, the true overall sensitivity of bone scanning has appeared to be only around 65%, suggesting that a significant amount of metastatic lesions may go undetected [27, 35]. Bone scan thus seems to systematically underestimate the burden of bone metastases in the primary staging of PCa and consequently performs insufficiently to reliably classify patients as oligometastatic [23, 35].

## PET-CT and PET-MRI

Recent developments in molecular imaging, in particular positron emission tomography (PET) using targeted PCa tracers, have demonstrated great promise in more accurate assessment of patients with oligometastatic disease [36]. The combination of metabolic PET imaging with morphological imaging in a hybrid PET-CT or PET-MRI exam has overcome the limitations of conventional imaging, particularly in the early detection of a limited number of metastases and in the setting of low PSA levels [35, 37]. Advantages of PET-MRI over PET-CT include less exposure to radiation and better correlation of a hot spot on PET with the intraprostatic lesion or changes in the bone marrow, but there is still poor clinical evidence of PET-MRI at this time [38]. In PCa, PET-CT has been investigated mainly for restaging of patients with biochemical recurrence after treatment but there is a growing body of evidence supporting its use in primary staging [35, 39, 40].

^18^F Fluoro-deoxy-glucose (FDG)-PET is the mainstay of molecular imaging and has demonstrated clinical usefulness in many tumors but has proven to be of limited value in PCa [31, 41]. Radiopharmaceutical agents showing a tropism for PCa cells such as ^11^C acetate, ^11^C choline and ^18^F choline have been introduced around the turn of the century, and, in the last 10 years predominantly choline-based tracers have been used in clinical practice [41–44]. While the early results were promising, the utility of choline PET-CT has been limited in the oligometastatic setting due to reduced sensitivity in patients with low PSA and in the early stages of metastatic spread [37]. Moreover, choline is not necessarily cancer-specific and it shows also uptake in areas of benign inflammation causing false-positive results in, for example, reactive lymph nodes [35]. The most promising data to date have been generated with radiotracers targeting the prostate-specific membrane antigen (PSMA) which is overexpressed in PCa cells [35, 45]. Initially reported in 2012, ^68^Ga PSMA HBED-CC PET-CT has shown to be superior to the older tracers predominantly owing to increased avidity of uptake and higher target-to-background ratio, resulting in an improved detection rate [45, 46]. Other emerging innovative PET radiotracers for PCa have been proposed such as ^18^F-Fluciclovine (FACBC), ^18^F-Bombesin, ^18^F-fluoro-5α-dihydrotestosterone (FDHT) and urokinase-type plasminogen activator receptor (uPAR), but these are almost exclusively being investigated in the setting of recurrent PCa or for visualization of the primary intraprostatic lesion but not for distant staging in primary PCa [41].

Regarding nodal staging in the primary setting, an extended pelvic lymphadenectomy still represents the gold standard and accurate imaging modalities are currently lacking. Metastatic lymph nodal spread is present in approximately 20–35% of the intermediate- and high-risk patients but they are the most common site of occult disease in de novo settings [46–48]. PET-CT has been shown to be superior as compared to conventional morphological CT or MRI in preoperative lymph node assessment [25, 49]. PET-CT using ^11^C choline or ^18^F choline tracers have shown a relatively good specificity of 89.5–99.7% and positive predictive value of 63.6–98.0% but only moderate sensitivity (ranging from 10 to 67%) for detecting involved lymph nodes [39, 41, 44, 49–54]. The sensitivity is higher in high-risk PCa patients (83.3%) than in the intermediate-risk group (33.3%) [49]. Choline PET-CT could thus provide helpful additional information in patients with suspected oligometastatic disease but since sufficient detection rates are not reached as compared to surgical lymph node dissection, its routine clinical use is not recommended at present to detect occult lymph node metastases in all patients with high-risk PCa in whom CT scan findings are normal [6, 50, 52, 53, 55].

**Fig. 2:**
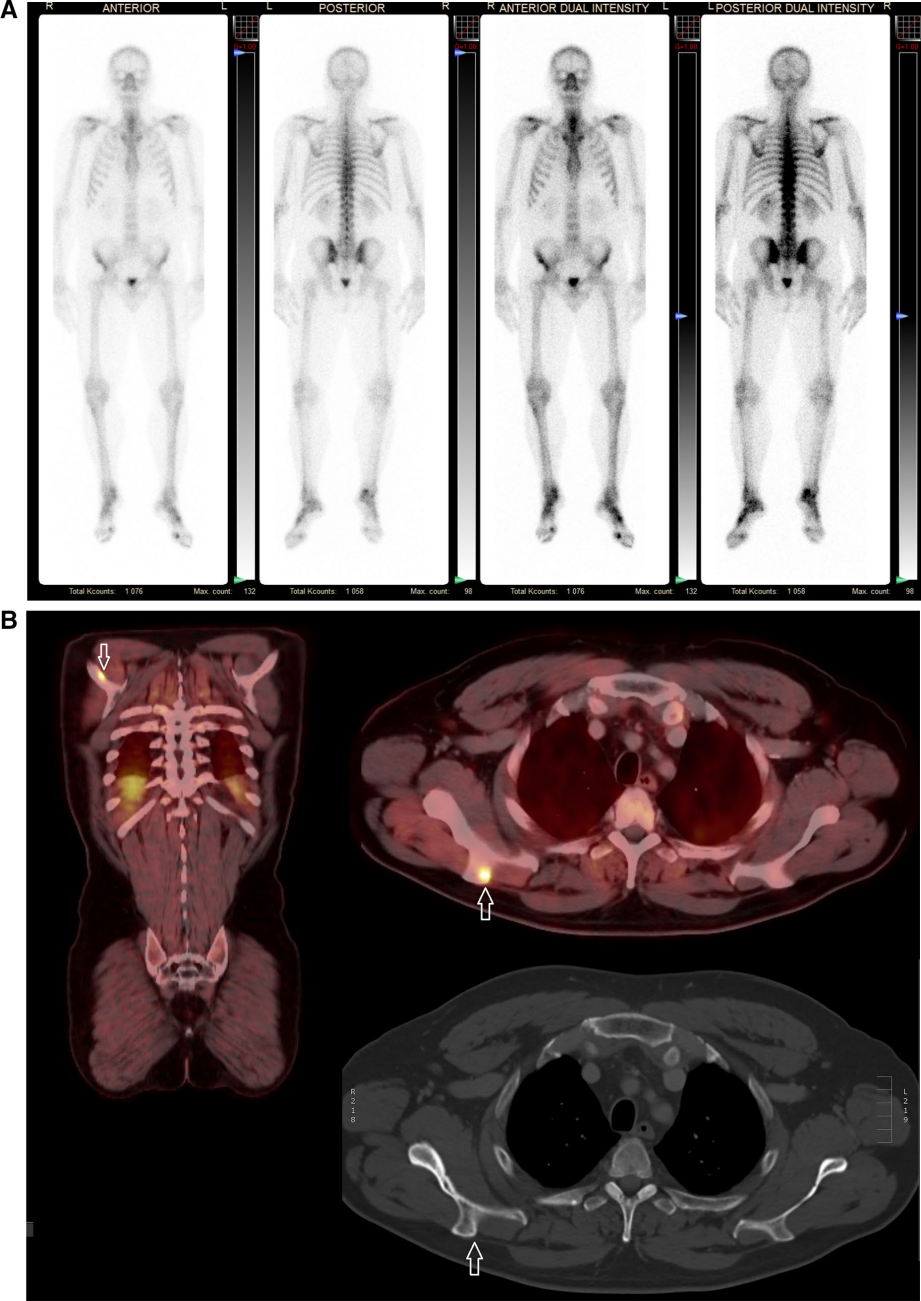
53-year-old man with a Gleason 4 + 4 PCa pT3 with PSA of 12.5 ng/ml. Primary staging with bone scan showed no lesions suspicious for bone metastases (**a**), but because he complained of right shoulder pain a ^18^F choline PET-CT was performed. This demonstrated focal tracer uptake in the spina scapulae (**b**, white arrow), indicating a bone metastase. On the corresponding CT image the scapula was unremarkable. Choline PET-CT may demonstrate bone metastases that are occult on bone scan and CT because it can detect changes in the bone marrow before osteoblastic reaction occurs. Nevertheless, bone scan currently remains the standard imaging modality in the primary staging of PCa

In addition, for the staging of bone metastasis, PET-CT appears to perform better than bone scan because tracer uptake may be detected in early bone marrow infiltration before osteoblastic reaction occurs (Fig. 2). A meta-analysis evaluating choline PET-CT in the diagnosis of bone involvement in intermediate or high-risk primary PCa patients showed a pooled sensitivity of 49% and a pooled specificity of 95% [31, 39, 51]. The poor sensitivity is largely due to non-recognition of early bone metastases, although choline or fluoride PET-CT detect bone metastases in up to 56% of patients with a negative or inconclusive bone scan [55, 56]. Nevertheless, because of the similar sensitivity, choline PET-CT should not replace bone scan to date.

**Fig. 3:**
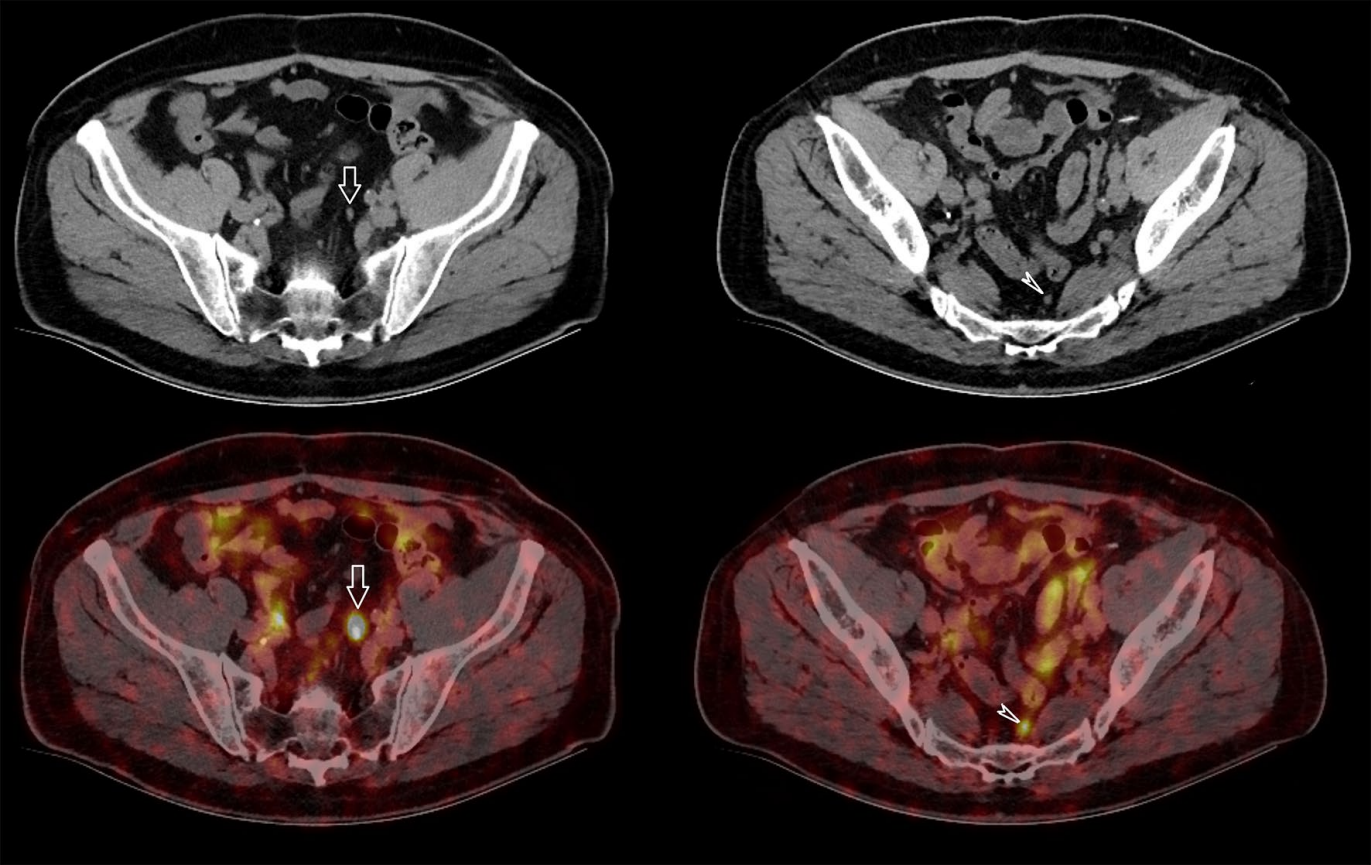
72-year-old man with biopsy proven PCa Gleason 4 + 3 pT2c and PSA of 6.7 ng/ml. Preoperative staging with imaging revealed two small lymph nodes in the pelvis, along the left common iliac artery (white arrow) and in the presacral fat (white arrowhead). On CT scan they both have a size of 5 mm which is below the morphological threshold to classify them as suspicious, but on ^68^Ga PSMA PET-CT they both showed highly avid tracer uptake, indicative of lymph node metastases. Pelvic lymph node dissection was performed and both lymph nodes proved to be malignant. Preoperative staging with ^68^Ga PSMA PET-CT thus appears to allow for more complete and accurate primary staging of PCa patients compared to standard routine imaging but its role in routine clinical practice is yet to be defined

^68^Ga PSMA PET-CT has currently been shown the best detection rates for the assessment of nodal, bone and visceral metastases, especially for smaller lesions at low PSA levels [37]. Gupta et al. [46] reported that in patients with newly diagnosed PC and high clinical suspicion of metastases (Gleason score 9–10 and/or serum PSA > 20 ng/ml) but nil or ≤ 3 metastases on conventional imaging (CT and bone scan) at least 1 lesion was detected in 100% of PSMA PET-CT. ^68^Ga PSMA PET-CT in primary staging found bone metastasis in 13% of asymptomatic patients with PSA < 10 ng/ml, although these patients would normally not have a bone scan in routine clinical practice [46]. In the systematic review of Perera [40] the pooled percentage of positive ^68^Ga PSMA PET-CT was 40% (95% confidence interval 19–64%) in the primary staging. Of all studies, 24% showed only oligometastases (≤ 3), and 62.5% of these concerned a solitary lesion. Moreover, a high rate of incidental extra-pelvic disease (41%) was observed [46]. Recently, another systematic review on the currently available data on ^68^Ga PSMA PET-CT in the primary staging of high-risk PCa was published [57]. A high variation in sensitivity (range 33–99%) was seen across the studies but with consistently high specificity (> 90%). Twelve studies were included but only five could be classified as high quality because they correlated imaging findings with histopathology. The lack of histologic correlation with positive (and indeed negative) PSMA PET-CT results makes it unclear as to the true accuracy of this novel imaging modality. Nonetheless, preoperative staging with ^68^Ga PSMA PET-CT appears to allow for more complete and accurate primary staging of PCa patients compared to standard routine imaging and may demonstrate a large number of otherwise unknown metastatic lesions, but its clinical role is yet to be definitively determined [40]. PET-CT has been reported to alter treatment plans in 29–76% of patients as compared to staging with conventional imaging [37, 39, 49, 56]. A large proportion of the additionally detected metastatic lesions are oligometastatic and in the future, with increasing use of ^68^Ga PSMA PET-CT, we may expect to identify a substantial higher number of patients with oligometastasis at initial presentation (Fig. 3). The clinical impact and cost effectiveness of potentially replacing current standard imaging for ^68^Ga PSMA PET-CT in the primary setting needs, therefore, to be confirmed by prospective studies with oncological outcomes, but despite this current lack of evidence, ^68^Ga PSMA PETC-CT seems to be increasingly performed in clinical practice in countries where it is available [37].

## Clinical implications

The rationale for using local treatment, as the first step in the management of de novo metastatic PCa, such as radical prostatectomy and radiotherapy is based on: a potential benefit in terms of improved local control, removal of the primary source for metastasis and (possibly) improved response to systemic treatments [58]. To this day, only retrospective population-based data, from different cancer registries such as Surveillance, Epidemiology, and End Results (SEER), National Cancer Database and several institutional data, support these assumptions. Despite the lack of level 1 evidence, approximately 75% of the panelists from the Advanced Prostate Cancer Consensus Conference (APCCC) 2017 agreed that there is a need for some form of local treatment in men with oligometastatic disease [59].

Although the results confirm the feasibility of cytoreductive RP, it is still unclear who would benefit from resection of the primary tumor. The ideal candidate might be a fit patient with ≤ T3 stage, Gleason score ≤ 7, low-volume metastatic disease, no visceral metastases and a low PSA nadir after 6 months of systemic therapy. Furthermore, imaging might be of use for selecting these candidates. The ongoing prospective randomized trials such as TRoMBone (ISRCTN15704862) and g-RAMPP (NCT02454543) will provide level 1 evidence regarding the role of surgery in patients with synchronous oligometastatic disease [60, 61].

A clinical decision based on the available data should be taken with caution due to significant limitations of these studies and unknown confounders: limited to no information regarding the associated comorbidities, different definitions of the oligometastatic state, different endpoints and unavailable data regarding the imaging techniques used for detection of distant lesions. Furthermore, data regarding treatments such as ADT, chemotherapy or dosage of radiotherapy are largely unavailable.

### Role of metastasis-directed therapies

Technical improvements in surgery and radiotherapy have introduced the option of metastasis-directed ablative therapies as an adjunct or alternative to standard-of-care systemic therapies [23]. More sensitive imaging will increase the likelihood of the detection of metastatic disease. However, none of the historic trials have incorporated any of these novel imaging modalities.

In a study by Sterzing et al., [62] 9 out of the 15 patients identified for primary staging by ^68^Ga-PSMA PET, were found to have metastatic lesions. Based on the results, approximately 51% of patients received a different therapeutic approach compared to the initial plan based upon conventional staging methods. Eight out of 15 patients were changed from M0 to M1a disease, which resulted in enlarged lymphatic field irradiation. These patients were all included in the historical trials as conventional imaging M0-status, whereas that with the current novel imaging techniques that the same number of patients was N1/M1 on novel imaging. Nevertheless, the disease control rates in these trials are already high [63, 64]. This begs the question whether change in management should be based on this novel imaging outside a trial as we have little evidence that metastasis-directed therapy influences a major endpoint in this setting.

### Future perspectives

In absence of more robust evidence from clinical trials, it is critical to be aware of how increasing availability and practical utilization of marker-based imaging in supposed non-metastatic disease, particularly PSMA PET-CT, with all its diagnostic accuracy may change treatment paradigms and de facto make decision-making process notably delicate nowadays, not least due to patients` wish. Since patients diagnosed with PCa increasingly obtain information on their disease on the Web, it is encouraging that quality, accessibility, and usability of the respective websites has been rated high in a contemporary analysis [65]. However, promising data on PSMA PET-CT exhibited there, are not ultimately accompanied by correct indications boosting demands for this imaging modality by laypersons even in disease settings without guideline recommendation for its use. The practice of performing PSMA PET-CT as primary staging in patients with high-risk localized or locally advanced disease has already become a reality in some scenarios. These patients would be treated with radical prostatectomy (± adjuvant radiotherapy) or primary radiotherapy + ADT based on the findings on conventional imaging (bone scan and CT) according to the EAU guidelines [2] but using PSMA PET-CT we would diagnose occult OPCa in some of them. Emerging dilemma is how this finding would influence our treatment strategy and how the cut-off for performing or not local treatment should be defined? Would we deprive patients of efficient local therapy eventually combined with currently still low-evidenced metastasis-directed therapy replacing it by escalated systemic treatment? Of note, long-term survival analysis of the CHAARTED Trial demonstrated a trend for patients with low-volume disease to perform even worse on chemohormonal treatment than on ADT (4% more deaths, p = 0.86) [66]. On the other hand, using PSMA PET-CT in some OPCa patients (as defined by conventional imaging) would screen out those with false-positive results, e.g., benign findings as bone hemangiomas, potentially shifting them to the cohort with clinically localized disease and enabling primary curative therapy. Until these issues are not sufficiently clarified by the results of solid clinical trials, staging of primary Pca in routine clinical practice should still be performed with conventional imaging (bone scan and CT), although we are aware of the limitations. In institutions where PSMA PET-CT is available, it could be used additionally in cases of suspected OPCa to confirm the limited number of metastatic lesions but then we are left to rely on empirical decisions and treatment strategy remains challenging.

## Conclusion

Ultimately, the decision regarding when and how to treat men with OPCa requires an assessment of the risks of burden of therapy versus risks of death derived from factors such as disease progression, associated comorbidities, metastatic load and access to medical facilities. With the recent data from the CHAARTED [67] and LATITUDE trials [68], an interesting question arises: is conventional imaging enough to accurately stage a patient in modern era? Functional imaging helps detect low-burden disease metastatic patients who are not likely to benefit from chemotherapy. However, these imaging modalities are not available in every center and thus clinicians may be prone to prescribe systemic treatment rather than referring patients for cytoreductive treatments. We hope that the ongoing prospective trials will help guide clinicians into making a more personalized management of synchronous metastatic patients.
